# Versatile Poly(Diallyl Dimethyl Ammonium Chloride)-Layered Nanocomposites for Removal of Cesium in Water Purification

**DOI:** 10.3390/ma11060998

**Published:** 2018-06-12

**Authors:** Sung-Chan Jang, Sung-Min Kang, Gi Yong Kim, Muruganantham Rethinasabapathy, Yuvaraj Haldorai, Ilsong Lee, Young-Kyu Han, Joanna C. Renshaw, Changhyun Roh, Yun Suk Huh

**Affiliations:** 1 Biotechnology Research Division, Advanced Radiation Technology Institute (ARTI), Korea Atomic Energy Research Institute (KAERI), 29, Geumgu-gil, Jeongeup-si, Jeonbuk 56212, Korea; scjscjsc@gmail.com (S.-C.J.); kgyong@kaeri.re.kr (G.Y.K.); islee2046@gmail.com (I.L.); 2 Department of Biological Engineering, Biohybrid Systems Research Center (BSRC), Inha University, 100, Inha-ro, Incheon 22212, Korea; sungmin.kang21@gmail.com (S.-M.K.); rmaannauniv@gmail.com (M.R.); 3 Radwaste Management Center, Korea Atomic Energy Research Institute (KAERI), 989, Daedeok-daero, Daejeon 34057, Korea; 4 Department of Energy and Materials Engineering, Dongguk University-Seoul, 30, Pildong-ro 1-gil, Seoul 04620, Korea; yuvraj_PD@yahoo.co.in (Y.H.); ykhan1972@gmail.com (Y.-K.H.); 5 Department of Civil and Environmental Engineering, University of Strathclyde, James Weir Building, 75 Montrose St, Glasgow G1 1XJ, UK; 6 Radiation Biotechnology and Applied Radioisotope Science, University of Science and Technology (UST), 217, Gajeong-ro, Daejeon 34113, Korea

**Keywords:** Prussian blue, magnetic nanoparticles, adsorbent, cesium, magnetic separation

## Abstract

In this work, we elucidate polymer-layered hollow Prussian blue-coated magnetic nanocomposites as an adsorbent to remove radioactive cesium from environmentally contaminated water. To do this, Fe_3_O_4_ nanoparticles prepared using a coprecipitation method were thickly covered with a layer of cationic polymer to attach hollow Prussian blue through a self-assembly process. The as-synthesized adsorbent was confirmed through various analytical techniques. The adsorbent showed a high surface area (166.16 m^2^/g) with an excellent cesium adsorbent capacity and removal efficiency of 32.8 mg/g and 99.69%, respectively. Moreover, the superparamagnetism allows effective recovery of the adsorbent using an external magnetic field after the adsorption process. Therefore, the magnetic adsorbent with a high adsorption efficiency and convenient recovery is expected to be effectively used for rapid remediation of radioactive contamination.

## 1. Introduction

Multifunctional polymer nanocomposites cover a very wide range of polymer matrices and hybrid polymer materials for their industrial applications [1,2].

The release of radionuclides, including fission products, from the long-term use of nuclear fuels and occasional nuclear disasters, such as at Chernobyl and the Fukushima Daiichi nuclear power plants, is an emerging issue [1,2,3,4]. In particular, radioactive cesium (^137^Cs), a fission product formed in nuclear reactors, is a problematic contaminant due to its high radioactivity, relatively long half-life (30.2 years), and bioavailability [5,6,7]. ^137^Cs can cause a number of significant health problems, including carcinoma of the kidney, liver, renal functions, and bladder as well as cardiovascular disease and gastrointestinal distress [8,9,10,11].

Various materials have been investigated for the removal of ^137^Cs, including polymers, zeolites, clay minerals, silica, and other nanomaterials [12,13,14]. However, these are unsuitable for column loading and are not easy to separate from aqueous solutions by filtration or centrifugation after cesium adsorption [4].

Prussian blue (PB) is a low-cost adsorbent with a strong affinity and high selectivity for cesium. It is also a U.S. Food and Drug Administration (FDA) approved drug employed for the handling of radioactive exposure [15] and has the potential for effective remediation of cesium. There are many reports on PB-based composites for the removal of radioactive cesium, such as PB/graphene foam [16], PB/silica [7,17], PB/glass pores [18], and PB/Alginate bead [19]. However, all of these adsorbents are either hard to synthesize or too expensive for large-scale applications. Magnetic separation, which has been used for the removal of cesium, is a fast, easy, and efficient method to recover an adsorbent. Combining PB and magnetic particles for the removal of radioactive cesium has many advantages. PB can selectively remove ^137^Cs and the adsorbent can be easily separated from environmentally contaminated water using an external magnet. 

In the present study, a novel nanocomposite material composed of PB/poly(diallyl dimethyl ammonium chloride) (PDDA)-coated magnetic particles (Fe_3_O_4_) was synthesized for efficient adsorption of ^137^Cs from an aqueous solution. The synthesized adsorbent was characterized using different techniques (e.g., XRD, FTIR, and TEM) and the effect of adsorbent dosage and pH on the removal efficiency of ^137^Cs was investigated. The developed adsorbent was recovered easily using an external magnet and showed a high adsorption capacity that can help solve the difficulty of recovering existing adsorbent materials.

## 2. Materials and Methods

*Materials*: Iron (II) chloride tetrahydrate (FeCl_2_·4H_2_O), Iron (III) chloride hexahydrate (FeCl_3_·6H_2_O), potassium hexacyanoferrate (III) trihydrate (K_3_[Fe(CN)_6_]·3H_2_O), PDDA solution (20 wt %, MW 400,000~500,000), hydrochloric acid (HCl) and ammonium hydroxide (NH_4_OH), polyvinylpyrrolidone (PVP, MW = 40,000) were purchased from Sigma-Aldrich, Korea. A standard cesium solution (1000 ppm) was purchased from Kanto Chemical Co. Inc. (Tokyo, Japan). All chemicals used in this study were of analytical reagent grade. Deionized (DI) water was used in this study. Additionally, ^137^Cs solution was provided from the Korea Atomic Energy Research Institute (KAERI), Daejeon, Korea.

### 2.1. Characterization

A transmission electron microscopy (TEM) analysis was performed using a Tecnai G2 (FEI, Eindhoven, The Netherland) microscope at an accelerating voltage of 200 kV and equipped with an energy dispersive spectrometer (EDS, Oxford Instruments, Oxford, UK). Fourier-transform infrared spectroscopy (FTIR) was analyzed using a Jasco-6600 spectrometer (JASCO, Weinheim, Germany). The hydrodynamic diameter and zeta potential measurements were carried out using a dynamic light scattering measurement collected on a Zetasizer Nano ZS (Malvern, UK). The X-ray diffraction (XRD) patterns were mainly collected using a Bruker D2 PHASER (Hamburg, Germany) diffractometer with Cu K(alpha) radiation. The Brunauer-Emmett-Teller (BET) surface area and average pore diameter were obtained from the N_2_ adsorption/desorption isotherm using a fully automatic physisorption analyzer (ASAP 2020, Micromeritics Instrument Corp., Norcross, GA, USA).

### 2.2. Preparation of Magnetic PDDA@Fe_3_O_4_

PDDA-coated magnetic particles were prepared according to a previous report [20]. A solution (20 mL) containing FeCl_2_·4H_2_O (0.8 g), FeCl_3_·6H_2_O (2.16 g), and PDDA (1.0%, *v*/*v*) was deoxygenated by bubbling with nitrogen gas for 10 min, followed by heating to 80 °C. Subsequently, 10 mL of 2% NH_4_OH was added quickly to the heated solution, which was left to stir for another 1 h. After cooling to room temperature, the obtained PDDA-coated Fe_3_O_4_ nanoparticles (NPs) were isolated using a magnet field and washed three times with deionized water. Finally, the nanoparticles were dried at comfortable room temperature.

### 2.3. Synthesis of Mesocrystal and Hollow PB Particles

For the synthesis of mesocrystal Prussian blue (MPB) [21], 3 g of PVP and 131.7 mg of K_3_[Fe(CN)_6_]·3H_2_O were added into a 0.1 M HCl solution (40 mL) under magnetic stirring, and a clear solution was obtained after 1 h. The solution was then heated in an oven at 80 °C for 20 h. The precipitate formed was then collected through centrifugation, washed with distilled water and ethanol two times, and dried at room temperature for 12 h. Hollow Prussian blue (HPB) particles were synthesized by controlled chemical etching of MPB particles [21]. In brief, 20 mg of MPB and 100 mg PVP were added to a 1.0 M HCl solution (20 mL) under magnetic stirring. After 1 h, the solution was transferred into a stainless autoclave and heated at 135 °C for 2.5 h. The HPB particles that were precipitated from the solution were subsequently collected by centrifugation, washed with distilled water and ethanol two times, and then dried at comfortable room temperature for 12 h.

### 2.4. Synthesis of PB@PDDA@Fe_3_O_4_ Composite

To synthesize the composite nanoparticles, 1 g of PDDA@Fe_3_O_4_ was mainly dispersed in 10 mL of distilled water, and 0.01 M HCl was added to adjust the pH to 6. Then, 3 g of MPB or HPB particles were added to the above solution and 0.01 M NaOH was added to adjust the pH to 6. The slurry obtained was thoroughly mixed at room temperature in a 50 mL polypropylene tube. The MPB or HPB particles were attached to the surface of PDDA@Fe_3_O_4_ due to the electrostatic interaction. The obtained HPB@PDDA@Fe_3_O_4_ composite was separated from the solution using an external magnet (1.4 teslas) and washed several times with distilled water.

### 2.5. Adsorption Experiments

The adsorption isotherms were investigated using batch experiments with a non-radioactive isotope, ^133^Cs. The initial Cs concentration varied from 1–200 ppm. Adsorbent (10 mg) was added to the Cs solution (4 mL). After equilibrating for 12 h, the adsorbent was separated by a magnet and the residual Cs concentration in the solution was analyzed by inductively coupled plasma mass spectrometry (ICP-MS, PerkinElmer ELAN6100, SCIEX PerkinElmer, Beaconsfield, UK). A radioactive ^137^Cs adsorption experiment is given in detail in the supporting information. All experiments were conducted 3 to 5 times.

### 2.6. Radioactive ^137^Cs Decontamination

Different amounts of HPB@PDDA@Fe_3_O_4_ composite (0.5, 2, or 5 mg) were added to 10 mL of the ^137^Cs aqueous solution (100 Bq/g); the concentrations of PB NPs/mL of the ^137^Cs solution were 0.05, 0.2, 0.5 mg/mL, respectively. After adsorption of ^137^Cs for 12 h, the adsorbent was removed using an external magnet. The solution concentrations of the ^137^Cs before and after treatment with the adsorbents were analyzed using a high-purity germanium (HPGe) detector (Canberra Inc., Meriden, CT, USA). In particular, we measured more than 3000 counts per second (cps) in each experiment for 1 h.

### 2.7. The Influence of pH on the Removal of ^137^Cs

The removal of ^137^Cs as a function of solution pH ranging from 4–10 was prepared by adjusting the pH using HCl and NH_4_OH. The initial ^137^Cs concentration was 100 Bq/g. All experiments were equilibrated for 12 h with stirring. The nanocomposite was then separated from the solution using a magnet, and the residual ^137^Cs concentration was measured using a HPGe detector (Canberra Inc., Meriden, CT, USA).

## 3. Results and Discussion

### 3.1. Fabrication of the Magnetic Adsorbent

**Figure 1:**
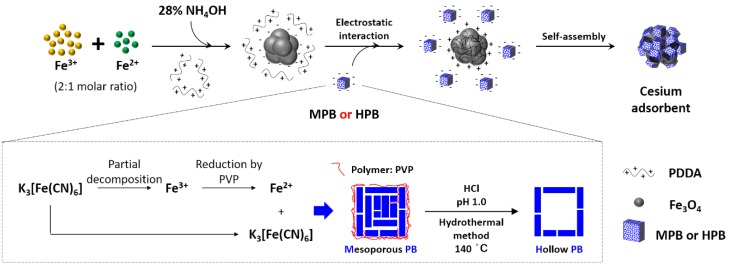
Schematic diagram of the synthesis of hollow Prussian blue (HPB)@ poly(diallyl dimethyl ammonium chloride) (PDDA)@Fe_3_O_4_ nanocomposites.

Figure 1 shows a schematic representation of the synthesis of PB@PDDA@Fe_3_O_4_. The PDDA@ Fe_3_O_4_ composite was prepared via a precipitation reaction. The negatively charged PB (MPB or HPB) was added in excess to a smaller amount of positively charged PDDA@ Fe_3_O_4_, and the surface area of the positively charged PDDA@Fe_3_O_4_ was completely covered by negatively charged PB via self-assembly.

**Figure 2:**
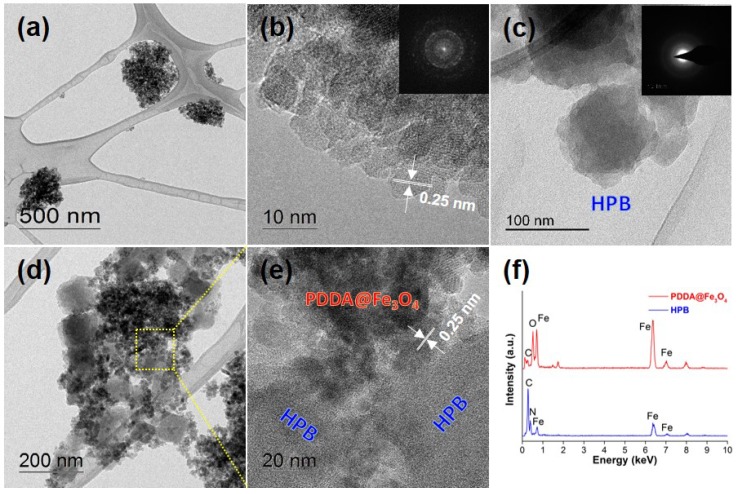
Representative (**a**) TEM and (**b**) HRTEM images of PDDA@Fe_3_O_4_ (The inset shows the fast Fourier transform (FFT) pattern of Fe_3_O_4_). (**c**) HRTEM of HPB nanoparticle (The inset shows selected area electron diffraction (SAED) pattern of HPB). Representative (**d**) TEM and (**e**) HRTEM images of the HPB@PDDA@Fe_3_O_4_ composite, (**f**) energy dispersive spectrometer (EDS) analysis of HPB and PDDA@Fe_3_O_4_ composite.

The TEM image of the PDDA@Fe_3_O_4_ composite showed aggregated particles with a mean diameter of 200–300 nm, and the subunits were smaller spherical nanocrystals with a size of about 10 nm (Figure 2a,b). HRTEM images (Figure 2b) of the subunits and their corresponding fast Fourier transform (FFT) patterns (Figure 2b inset) indicated the presence of Fe_3_O_4_; the spacing of the lattice planes were calculated to be 0.25 nm, which corresponds to the families of crystal planes of Fe_3_O_4_. The FFT pattern and spacing of the lattice planes showed exactly the same diffraction patterns compared to Fe_3_O_4_ (Figure S1).

Two forms of PB NPs were used: MPB and HPB. MPB was achieved by direct dissociation of a single-source precursor K_3_[Fe(CN)_6_] with PVP as a capping and reducing agent. The whole reaction was based on the partial decomposition and reduction of K_3_[Fe(CN)_6_] in acidic solution with the addition of PVP. [Fe(CN)_6_]^3−^ ions were slowly dissociated into Fe^3+^ ions in acidic solution and the Fe^3+^ ions were subsequently reduced into Fe^2+^ ions by the weak reducer (PVP). Finally, Prussian blue was synthesized through the recrystallization of small particles by the assistance of PVP. MPB was chemically etched with HCl to create HPB with interior hollow cavities. The surface of the HPB (Figure S2c,d) was much rougher than MPB (Figure S2a,b) and the particles size was smaller because of chemical etching. When H^+^ ions diffused into the MPB, the local concentration of H^+^ ions in the center part of the MPB was higher than that of the particle surface. The etching rate of Prussian blue in the center became relatively high, thereby reshaping the interior hollow pore. The TEM image of the HPB (Figure 2c) displayed a network of connected particles. The corresponding selected area electron diffraction (SAED) pattern (Figure 2c inset) showed a diffused ring pattern, indicating a polycrystalline nature [22]. Figure 2d,e shows that the HPB particles were successfully decorated onto the surface of the PDDA@Fe_3_O_4_ composite. The coating of PDDA onto the Fe_3_O_4_ particles provided a positively charged surface, which could combine easily with the HPB nanoparticles because of their electronegative nature (Figure 2e). The EDS spectra (Figure 2f) of a bright field TEM image revealed that the nanocomposite consisted of Fe and O in Fe_3_O_4_ area and Fe, C, and N in the HPB area (as indicated in Figure 2e).

### 3.2. Morphological and Surface Studies

**Figure 3:**
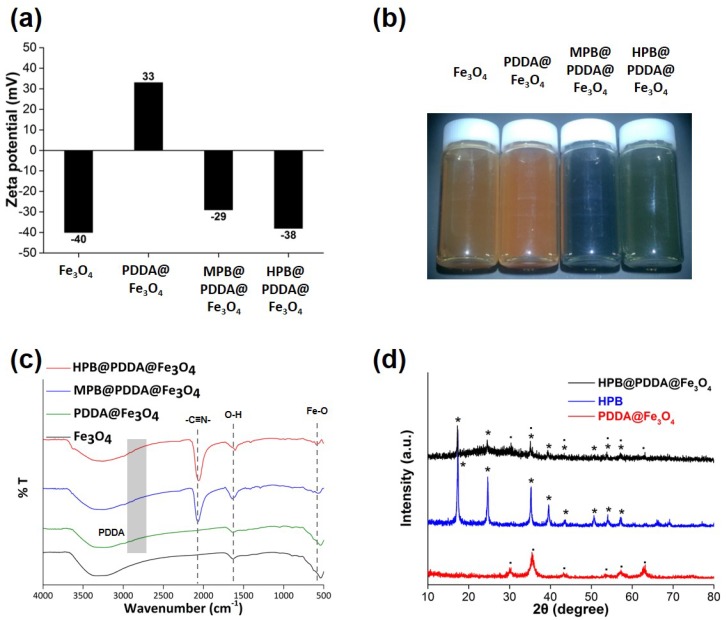
Representative (**a**) Zeta potential values; (**b**) Optical image; (**c**) Fourier-transform infrared spectroscopy (FTIR) spectra; and (**d**) XRD patterns of Fe_3_O_4_, PDDA@Fe_3_O_4_, mesocrystal Prussian blue (MPB)@PDDA@Fe_3_O_4_, and HPB@PDDA@Fe_3_O_4_.

The surface coating also affected the zeta potential and particle size. The zeta potential values of the Fe_3_O_4_, PDDA@Fe_3_O_4_, MPB@PDDA@Fe_3_O_4_, and HPB@PDDA@Fe_3_O_4_ in water were −40, 33, −29 and −38 mV, respectively (Figure 3a). Figure 2b exhibits a photographic image of an aqueous solution of Fe_3_O_4_, PDDA@Fe_3_O_4_, MPB@PDDA@Fe_3_O_4,_ and HPB@PDDA@Fe_3_O_4_. After the reaction between Fe_3_O_4_ and PB, the aqueous solution turned a blue or dark green, indicating PB on the surface of the magnetic nanoparticles (MNPs).

The FTIR spectra also demonstrated the successful coating of MPB or HPB on the PDDA@Fe3O4 surface (Figure 3c). The absorption bands at 3375–3787 and 1615 cm^−1^ refer to the O–H stretching mode and H–O–H bending mode due to water molecules. The prominent peak intensity of PB at 2076 cm^−1^ was observed in HPB@PDDA@Fe3O4 (Figure 3c). The common characteristics of PB were observed from the absorption band at 2076 cm^−1^ owing to the stretching vibration of the C≡N group [22]. For the PDDA sample, a strong band at ~3440 cm^−1^ was attributed mainly to –NR_3_^+^ stretching vibration, and the bands around the 3000–2800 cm^−1^ region were assigned to the C–H bending, as shown in Figure 3c [23]. The presence of Fe–O bond from Fe_3_O_4_ was also able to be seen at 539 cm^−1^ for all those samples with Fe_3_O_4_. From the FTIR results, the presence of both Fe_3_O_4_ and HPB were identified for all samples.

Figure 3d shows the XRD patterns of the samples. For PDDA@Fe3O4, all the diffraction peaks were in good agreement with the face-centered cubic (fcc) structure of magnetite corresponding to (220), (311), (400), (422), (511), and (440) (Figure S3). After HPB coating, a new diffraction peak was shown at 17.4°, 24.8°, and 35.3°, which were addressed to (200), (220), and (222) reflections, respectively, of the fcc structure of the HPB nanoparticles.

**Figure 4:**
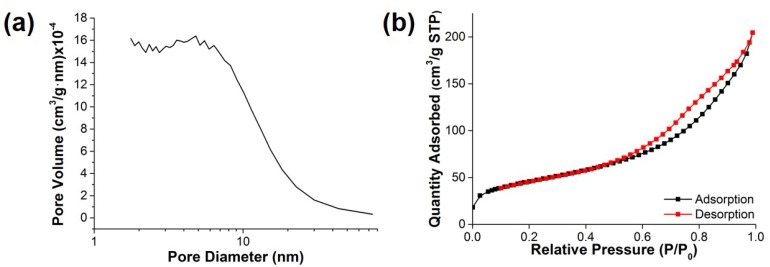
Representative (**a**) pore size distribution and (**b**) N_2_ adsorption/desorption isotherm of the HPB@PDDA@Fe_3_O_4_ composite.

Figure 4 shows the N_2_ adsorption/desorption isotherm and corresponding Barrett–Joyner–Halenda (BJH) pore size distribution curve of the HPB@PDDA@Fe_3_O_4_ composite. The pore volume of the HPB@PDDA@Fe_3_O_4_ ranged from 2 to 10 nm (Figure 4a). The isotherm exhibited that the BET specific surface area of the composite was 166.16 m^2^/g (Figure 4b).

### 3.3. Performance Evaluation of Cesium Removal

**Figure 5:**
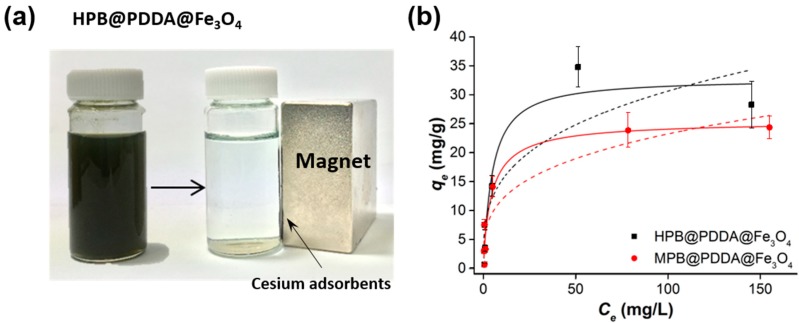
(**a**) Photograph shows the recovery of the composite with an external magnet after Cs adsorption; (**b**) nonlinear Langmuir (solid lines) and Freundlich (dotted lines) isotherm models.

The uptake of cesium by MPB@PDDA@Fe_3_O_4_ and HPB@PDDA@Fe_3_O_4_ composites was investigated in the batch experiments. After 12 h of contact, the magnetic composites were easily collected to one side of the vial by positioning a magnet against the vial (Figure 5a). The NPs were rapidly separated within 30 s. This demonstrated that the adsorbent could be very easily recovered using an external magnet. The adsorption capacity of magnetic cesium adsorbents was investigated using Langmuir and Freundlich adsorption isotherm models. The Langmuir model [24] is based on the assumption that all active sites are independent and equivalent, and it indicates a monolayer adsorption process for cesium onto the uniformly adsorbent surface. The nonlinear forms of the equation are written as:

(1) $$q_{e}\;=\;q_{max}\frac{K_{L}C_{e}}{1+K_{L}C_{e}}$$

where *q_e_* and *q_max_* are the equilibrium adsorption capacity and monolayer maximum adsorption capacity (mg/g), respectively, and *K_L_* is a constant related to the affinity between the adsorbent and the adsorbate. *C_e_* is equilibrium concentrations of inactive cesium in the solution. By comparison, the Freundlich adsorption isotherm model [25] is significantly considered to be an empirical equation that describes multilayer adsorption with several types of adsorption sites on the surface of an adsorbent. The model consists of the following equation:

(2) $$q_{e}\;=\;K_{F}C_{e}^{\frac{1}{n}}$$

where *K_F_* and n are the Freundlich constants relative to the multilayer adsorption capacity. The experimental data of HPB@PDDA@Fe_3_O_4_ and MPB@PDDA@Fe_3_O_4_ composites were a better fit to the Langmuir model (R^2^ = 0.91 and 0.90, respectively) than the Freundlich model (R^2^ = 0.79 and 0.86, respectively). The MPB@PDDA@Fe_3_O_4_ and HPB@PDDA@Fe_3_O_4_ possessed a *q_max_* of 25.6 and 32.8 mg/g, respectively (Figure 5b).

### 3.4. Radioactive Cesium Adsorption Studies

**Figure 6:**
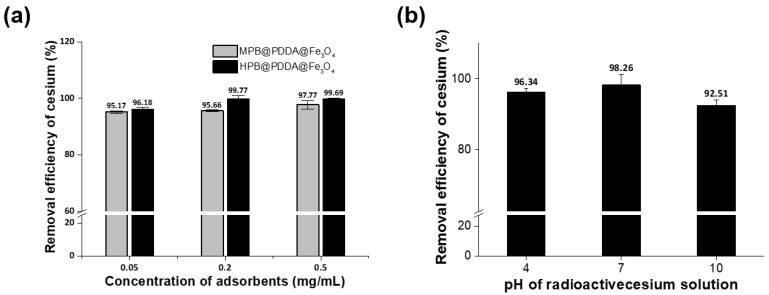
(**a**) Removal efficiency of ^137^Cs by MPB@PDDA@Fe_3_O_4_ and HPB@PDDA@Fe_3_O_4_ composites and (**b**) influence of pH on the uptake of ^137^Cs by HPB@PDDA@Fe_3_O_4_ composite.

Figure 6a shows the efficiency of adsorbents for the removal of ^137^Cs from an aqueous solution using different adsorbent concentrations (0.05, 0.2, and 0.5 mg/mL). The initial ^137^Cs concentrations were 92.38 Bq/g and 86.44 Bq/g for MPB- and HPB-coated nanoparticles, respectively. For a MPB@PDDA@Fe_3_O_4_ composite, the removal of ^137^Cs from the solution per gram of adsorbent was increased from 95.17% to 97.77% when increasing the adsorbent concentration from 0.05 to 0.5 mg/mL. In the case of the HPB@PDDA@Fe_3_O_4_ composite, the removal efficiency increased from 96.18% to 99.69% with an increase in adsorbent concentration. This higher removal efficiency probably results from the higher surface area of the HPB@PDDA@Fe_3_O_4_ compared to the MPB@PDDA@Fe_3_O_4_. The effect of pH on ^137^Cs uptake by the HPB@PDDA@Fe_3_O_4_ composite was investigated, as shown in Figure 6b. At the three pH values investigated (pH 4, 7, and 10), the HPB@PDDA@Fe_3_O_4_ adsorbent removed over 92% of ^137^Cs, and a maximum ^137^Cs uptake of 98.26% was achieved at pH 7. When the pH further increases or decreases, the adsorption sites become available for cation or anion in ion exchange processes, which leads to difficult cesium adsorption. Even though the removal efficiency of cesium ions is feasible across a wide range of pH values, the best results are achieved at neutrality.

The distribution coefficient (*K_d_*) was defined to analyze the cesium removal ability and adsorption performance of HPB@PDDA@Fe_3_O_4_ toward ^137^Cs:

(3) $$K_{d}\;=\;\frac{C_{0}-C_{f}}{C_{f}}\times \frac{V}{M}$$

where *C*_0_ and *C_f_* are the initial and final concentrations of Cs in the solution before and after equilibrium contact with the adsorbent, V is the volume of the solution, and M is the mass of the adsorbent used. For the HPB@PDDA@Fe_3_O_4_ composite, the *K_d_* was calculated as ~6.4 × 10^5^ mL/g, which was an order of magnitude higher than the literature value for the PB adsorbent of 5.2 × 10^4^ mL/g [7]. Based on these results, we speculate that the resulting hollow adsorbents provide a wider reaction space through which cesium can be adsorbed, thereby increasing the adsorption capacity.

## 4. Conclusions

In this study, we successfully synthesized a new adsorbent material that shows a high cesium adsorption efficiency and easy recovery of adsorbents by decorating hollow PB onto a magnetic PDDA@Fe_3_O_4_ composite. The hollow PB attached to the outside of the magnetic particles—like a bunch of grapes—can increase the active specific area and consequently improve the cesium adsorption capacity. Furthermore, the excellent magnetic composite property makes it possible to recover the adsorbent effectively and selectively bind cesium from contaminated radioactive wastewater through a magnetic field. In the selective adsorption experiment, the adsorbent, HPB@PDDA@Fe_3_O_4_, exhibited an excellent maximum adsorption capacity of 32.8 mg/g and ^137^Cs removal efficiency of 99.69%. This new approach for the synthesis of a functional adsorbent can be an important contribution towards the effective removal of ^137^Cs.

## Supplementary Materials

The following are available online at http://www.mdpi.com/1996-1944/11/6/998/s1. Figure S1: (a–c) TEM images of Fe3O4 nanoparticles, Figure S2: (a) TEM image of MPB and (b) magnified image. (c) TEM image of HPB and (d) magnified image, Figure S3: XRD patterns of Fe3O4 nanoparticles.
