# Supplementary material for: Versatile Poly(Diallyl Dimethyl Ammonium Chloride)-Layered Nanocomposites for Removal of Cesium in Water Purification
Source: Materials (Basel). 2018 Jun 12;11(6):998. doi: 10.3390/ma11060998 (PMC6025151; doi:10.3390/ma11060998)
Supplement: Supplementary file 1 [file materials-11-00998-s001.pdf]

Supplementary Materials: Versatile Poly(diallyl dimethyl ammonium chloride)-Layered Nanocomposites for Removal of Cesium in Water Purification

Sung-Chan Jang, Sung-Min Kang, Gi Yong Kim, Muruganantham Rethinasabapathy, Yuvaraj Haldorai, Ilsong Lee, Young-Kyu Han, Joanna C. Renshaw, Changhyun Roh, Yun Suk Huh

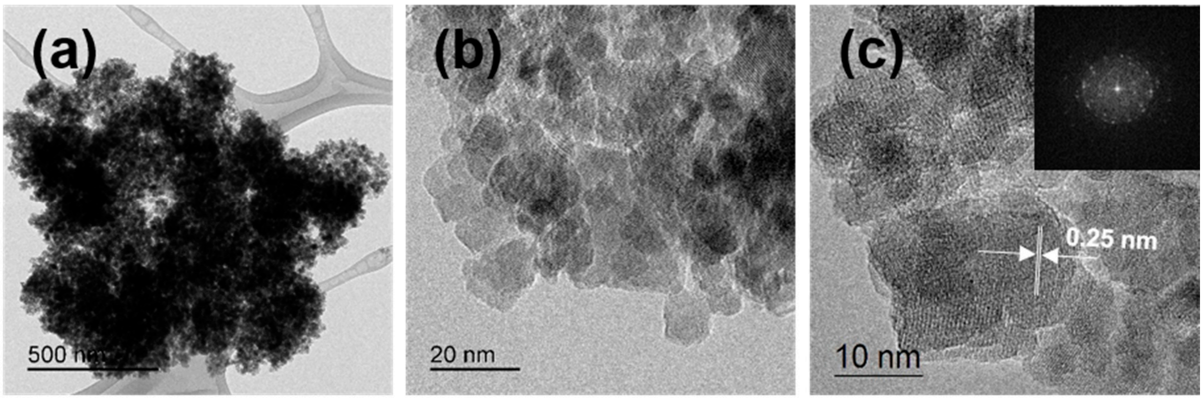

Figure S1. (a-c) TEM images of Fe_3O_4 nanoparticles.

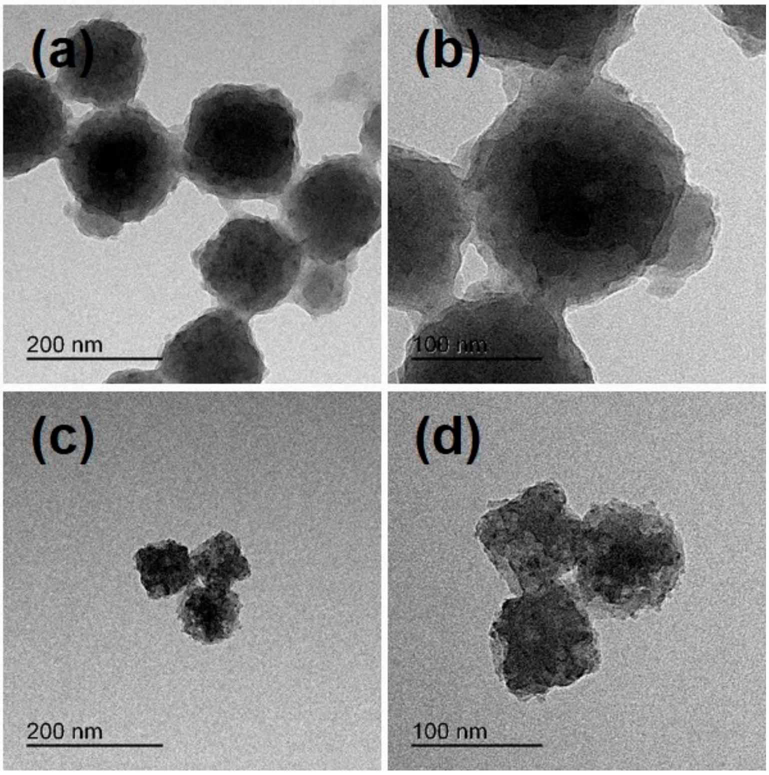

Figure S2. (a) TEM image of MPB and (b) magnified image. (c) TEM image of HPB and (d) magnified image.

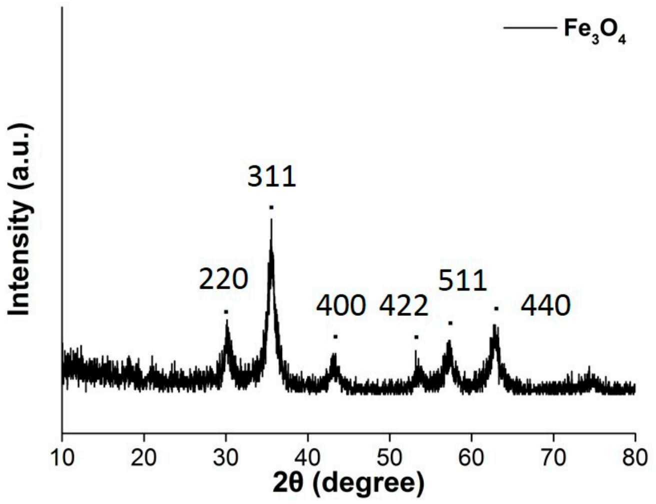

Figure S3. XRD patterns of Fe_3O_4 nanoparticles.
